# RETRACTED: Bas et al. Embedded Sensors with 3D Printing Technology: Review. *Sensors* 2024, *24*, 1955

**DOI:** 10.3390/s24247957

**Published:** 2024-12-13

**Authors:** Joan Bas, Taposhree Dutta, Ignacio Llamas Garro, Jesús Salvador Velázquez-González, Rakesh Dubey, Satyendra K. Mishra

**Affiliations:** 1 Space and Resilient Communications and Systems (SRCOM), Center Technologic de Telecomunicacions de Catalunya (CTTC), Avinguda Carl Friedrich Gauss, 11, 08860 Castelldefels, Spain; joan.bas@cttc.es; 2 Department of Chemistry, Indian Institute of Engineering Science and Technology, Shibpur, Howarh 711103, India; taposhreedutta812@gmail.com; 3 Navigation and Positioning, Center Technologic de Telecomunicacions de Catalunya (CTTC), Avinguda Carl Friedrich Gauss, 11, 08860 Castelldefels, Spain; illamas@cttc.es (I.L.G.); jvelazquez@cttc.es (J.S.V.-G.); 4 Institute of Physics, University of Szczecin, Wielkopolska 15, 70-451 Szczecin, Poland; rakesh.dubey@usz.edu.pl

The *Sensors* Editorial Office retracts the article, “Embedded Sensors with 3D Printing Technology: Review” [1], cited above.

Following publication, concerns were brought to the attention of the Editorial Office regarding an overlap between this publication [1] and a previously published article [2] produced by different authors.

Adhering to our complaints procedure, the Editorial Office and Editorial Board conducted an investigation that confirmed a significant overlap between these two articles [1,2], without appropriate acknowledgment or citation. As a result, the Editorial Board have decided to retract this paper as per MDPI’s retraction policy (https://www.mdpi.com/ethics#_bookmark30).

This retraction was approved by the Editor-in-Chief of *Sensors*.

The authors did not agree with this retraction.
